# Application of Machine Learning for Fenceline Monitoring of Odor Classes and Concentrations at a Wastewater Treatment Plant

**DOI:** 10.3390/s21144716

**Published:** 2021-07-09

**Authors:** Federico Cangialosi, Edoardo Bruno, Gabriella De Santis

**Affiliations:** Tecnologia e Ambiente (T&A), 8-70017 Putignano, Italy; e.bruno@tecnologiaeambientesrl.com (E.B.); g.desantis@tecnologiaeambientesrl.com (G.D.S.)

**Keywords:** electronic nose, instrumental odor monitoring system, odor classification, odor quantification, multilayer perceptron, random forest

## Abstract

The development of low-cost sensors, the introduction of technical performance specifications, and increasingly effective machine learning algorithms for managing big data have led to a growing interest in the use of instrumental odor monitoring systems (IOMS) for odor measurements from industrial plants. The classification and quantification of odor concentration are the main goals of IOMS installed inside industrial plants in order to identify the most important odor sources and to assess whether the regulatory thresholds have been exceeded. This paper illustrates the use of two machine learning algorithms applied to the concurrent classification and quantification of odors. Random Forest was employed, which is a machine learning algorithm that thus far has not been used in the field of odor quantification and classification for complex industrial situations. Furthermore, the results were compared with commonly used algorithms in this field, such as artificial neural network (ANN), which was here employed in the form of a deep neural network. Both techniques were applied to the data collected from an IOMS installed for fenceline monitoring at a wastewater treatment plant. Cohen’s kappa and Normalized RMSE are used as specifical performance indicators for classification and regression: the indicators were calculated for the test dataset, and the results were compared with data in the literature obtained in contexts of similar complexity. A Cohen’s kappa of 97% was reached for the classification task, while the best Normalized RMSE, namely 4%, for the interval 20–2435 ouE/m^3^ was obtained with Random Forest.

## 1. Introduction

Odors are a relevant component of atmospheric pollution and an important indicator of the health of urban areas close to urban wastewater treatment plants (WWTPs) [1]. As a result of the continuous expansion of cities, these plants are located closer to residential receptors, sometimes at distance of less than 500 m to 1 km. Moreover, urban WWTPs in the EU are not subject to the Industrial Emission Directive (IED) 2010/75/EU or the related Best Available Techniques to avoid the emission of odors, nor have maximum allowable odor concentrations been established. Although no legislation regulating odor, with specific requirements on odor emissions from sources, is in force in Europe, America, or Australia, various regions within certain member states in the EU have implemented their own regulations in order to define discharge limits from sources and how odor pollution issues for urban WWTPs should be managed [2,3,4].

In such a complex and socially sensitive context due to the various reports of bad odors in the proximity of urban WWTPs, the contribution of citizen science for identifying odor emissions [5,6,7] and the electronic nose or Instrumental Odor Monitoring System (IOMS) [8] are powerful tools to help policy makers and environmental protection agencies define appropriate strategies or specific prescriptions on a case-by-case basis in order to identify, measure, and reduce the impact of odors on receptors.

IOMS offer advantages over dynamic olfactometers because they are designed for real-time measurements [9,10] and do not suffer from the limitations of citizen science approaches, such as biased information and high variability of results [11]. Furthermore, the rapid development of low-cost sensors, which allows for air pollution monitoring at a lower cost and with a higher spatial density than reference measurement methods, the work undertaken by the Technical Committee CEN/TC 264 “Air quality” and the relative working group (WG41) for defining technical specifications for the performance evaluation of air quality sensors, and the development of the high-speed communications network (5G) are the most significant recent developments for the field of IOMS hardware. The software developments in recent years are remarkable both in the field of data pretreatment and feature extraction [12,13], and algorithms for odor classification [14,15] and quantification [16]. Several machine learning (ML) algorithms were proposed for pattern recognitions of odor, such as PCA, LDA, ANN, PLS, and SVM, and these were employed as regression techniques in order to obtain odor concentrations [17,18,19]. However, most software developments, including the recent use of Random Forest classification [20], were achieved in the laboratory or in controlled situations, where the environmental conditions (relative humidity and temperature) relevant in the environmental monitoring of odor emissions were not considered.

On the other hand, performance validation of IOMS for odor classification and concentration analyses for complex situations with several odor sources have been carried out in the last few years with promising results. This enables valuable information to be gathered on the time variations of odors from the sources [21,22,23]. However, in such case studies, little detail is provided on the algorithms employed for odor classification and regression, and the performance indicators for classification and regression are not extensively discussed. In other words, in the literature, there are several studies in which ML algorithms for odor classification and quantification are discussed and compared in controlled situations or for the purposes of production quality control [24,25,26]; however, in the field of environmental monitoring applications, such information is lacking, and only the direct comparison of the results of commercial IOMS with real data is shown [27], without any information on the algorithms employed to derive the measurements. In the near future, information on the algorithms employed, feature selection, training procedures, cross-validation, and test set-related performance indicators could be essential when a commercial or prototype IOMS is installed in the field, which are far more relevant for the final measurement than the sensor characteristics. Moreover, field IOMS applications for environmental monitoring are discussed in the literature for environmental monitoring of either odor concentrations only or odor classification only, with few simultaneous monitoring applications [22]. Moreover, the ones that exist lack detailed information on the algorithms used. Only recently has an application of IOMS in complex industrial plants with qualitatively and quantitatively different emission sources been thoroughly discussed with reference to the ML algorithms employed for concurrent odor classification and quantification [28].

The main goal of this work was to describe a field IOMS application for the simultaneous monitoring of odor concentrations and classes in an urban WWTP, in which the ML algorithms employed are described in detail, with reference to Random Forest, which thus far has not been used in the field of odor quantification and classification for complex industrial situations. In detail, as part of a project for monitoring odor emissions from urban WWTPs and the related sources [29], a dataset of odor classes and concentrations was created, consisting of samples collected by different sources of odor emissions from WWTPs, each with time-varying concentrations. This research discusses training and testing of an IOMS coupled with two Machine Learning algorithms developed for the purpose: an artificial neural network (Multilayer Perceptron, MLP) and Random Forest (RF), the former having already been employed for IOMS operating for fenceline odor monitoring [30,31], while the latter has yet to be used for odor classification or regression in environmental applications. Both techniques were applied to the data collected from an IOMS installed for fenceline monitoring at a WWTP. The results obtained from the models are discussed, and performance parameters for classification and regression were identified and quantified.

## 2. Materials and Methods

### 2.1. Odor Emission Source Characterization

**Figure 1:**
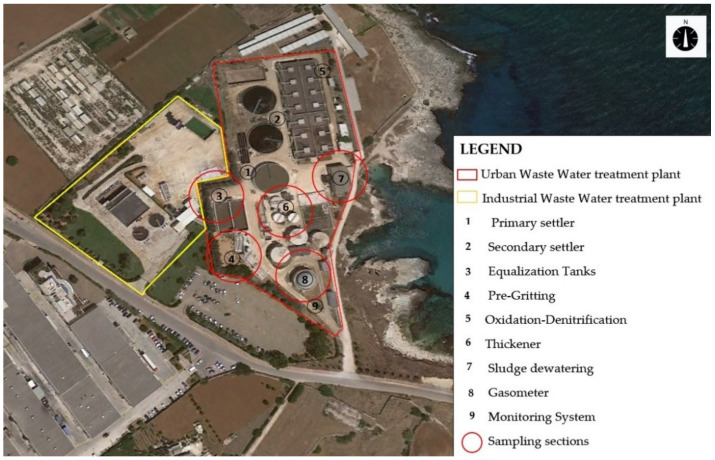
WWTP of Monopoli, indicating the sampling points.

The urban WWTP here considered is located in the industrial area of Monopoli (Bari–Italy), which is designed for a 64,695 population equivalent (PE) and operating with an average daily flow of 12,939 m^3^/d (Figure 1). The plant is located at a distance of 420 m from the closest residential buildings.

On the basis of the data from various studies carried out for the odor characterization of WWTPs [9,32], it was established that the most critical sections are pretreatments, primary sedimentation, and sludge conditioning. On the basis of such studies and evidence gathered in the field, WWTP odor mapping was carried out: the first phase comprised a complete characterization of the emission sources, and subsequently, a sampling program of the most critical sources was defined. The sampling program for collecting samples for training and testing was designed in order to take into account environmental variations in the ambient air (temperature, relative humidity). Thus, it lasted several months: from October to April.

As a result of regional enforcement [4,33], the sections of pretreatment and sludge thickening were covered, depressurized, and the air was treated in two biofilters; moreover, an anaerobic digester for sludge was active, which was associated with energy recovery by combustion, so that even biogas may be an important source of fugitive emissions.

After preliminary characterization of the source emissions, it was established that the most critical emission sources were pretreatments, sludge conditioning, and biogas from the anaerobic digester. The selected odor classes were Class 1 (pretreatments), Class 2 (sludge conditioning), Class 3 (biogas), and Class 0, defined as “ambient air”, which was not influenced by the above emission sources. As pretreatments and sludge conditioning reactors are enclosed and set in a depression, samples were taken from inlet pipelines to the biofilters and from the stacks, while biogas samples were taken from the pipeline connecting the anaerobic digester to the energy recovery section. The samples of Class 0 were collected in the WWTP area, taking care that the air at the moment of sampling was not affected by the odor sources considered in the study; however, as a result of the proximity of another industrial wastewater treatment plant, it was not always possible to sample odorless air. In Figure 1, the locations of the sampling points are defined.

Air samples were collected in accordance with EN 13725:2003, where Nalophan^®^ sampling bags with an 8 L volume were used; the samples with higher concentrations were diluted to 1:10 to increase the dataset size with a predilution system: model EPD10000 (Olfasense GmbH, Kiel, Germany). The IOMS (MSEM32^®^ by Sensigent, Baldwin Park, CA, USA) was located in position 9 (Figure 1), near the fenceline of the plant along the direction of prevailing winds coming from the plant to the nearest urban receptors. After collection in duplicates, each sample was fed to the IOMS on the same day, and the twin sample was analyzed using dynamic olfactometry (DO) at the T&A Laboratory within 24 h, using the LEO dynamic olfactometer (ARCO Solutions srl, Trieste, Italy) for the measurement of Odor Concentrations (Cod), which are expressed as European odor unit (ouE/m^3^). A total of 51 samples were collected, with odor concentrations ranging from 20 to 2435 ouE/m^3^.

### 2.2. Experimental Protocol for Data Acquisition

The dataset used for ML application was collected by an IOMS with an array of 32 sensors (S3, S4, S5 Environmental Sensors, i.e., pressure, humidity, and temperature, S7, S8, S9, S10 Electrochemical Sensors, S11-12-13-14 Metal Oxide Sensors, S18 Photoionization Detector, S21-22-23-24 Nanocomposite-based Sensors, S42-43-44-45-46-47-48-49 Metal Oxide Sensors, S50-51-52-53-54-55 Nanocomposite-based Sensors, S56-57 Metal Oxide Sensors). The instrument was equipped with a cartridge filter to obtain odorless air (zero air-ZA): air was drawn by the instrument after filtering to remove odorous compounds but preserve the temperature and humidity of the ambient air. The training phase was carried out in the field, as it is important that the samples were fed in different environmental conditions (humidity and temperature) [34], as variations in temperature and humidity may significantly influence the baseline of resistance of the sensors [35,36] and, consequently, affect the calculations of the sensor responses. In order to ensure that the baseline before sample feeding represented the ambient air conditions of odorless air, ZA was drawn in by the instrument. Moreover, a certain amount of time elapsed before the following feeding so that the sensors returned to the baseline values. Consequently, the measurement cycle consisted of 1 min of ZA, 2 min sample draw-in by IOMS (acquisition phase), and 3 min for baseline recovery; for each sample, the measurement cycle was repeated four times. The duration of each phase was determined on the basis of the literature [13] and observing the response of the sensors in terms of the time required to reach a steady state value during the feeding of the samples, which was less than 90 s (see Section 2.3). The sampling frequency of the instrument was set to 0.1 Hz, so that 12 data points were available for each sample replicate.

### 2.3. Data Pretreatment

**Figure 2:**
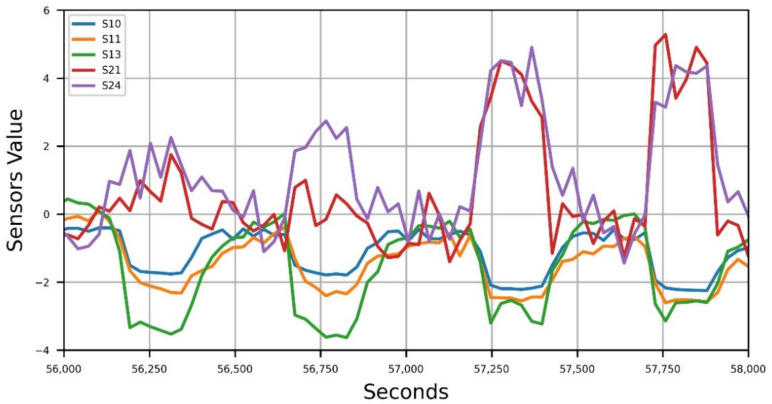
Measurements cycles during sample feeding.

All data collected by the IOMS, representing the sensor responses, were stored in a secure cloud storage and then extracted for elaboration. In Figure 2, the selected sensor responses during the four measurement cycles of one sample are shown, in which a simple zero normalization was applied to the process data for better visualization.

Data smoothing using low-pass digital filters was useful to clean up the signals and better identify the response of the sensors. A Savitzky–Golay filter based on least-squares smoothing with a polynomial order of 3 was used to filter the data, as this algorithm reduces the noise while maintaining the shape and height of the original signal [37,38]. Then, the response curves, representing the dynamic variation in the electrical resistance of the sensors [39], were analyzed to extract piecemeal signal features. Although several features are extractable from the response curves (steady-state response, rising time, falling time) [12], recent studies suggest that the peak values of the signals, representing the maximum degree of change in the sensors responding to odor, are more effective for odor classification [13].

Consequently, for the sensor *i*, the fractional difference *x_i_* in the signal relative to the peak values was used [12,13], by considering the associated piecemeal signal within the 2-min acquisition phase:

(1) $$x_{i}=\left|R_{i}-R_{bl,i}\right|/R_{bl,i}$$

where *R_i_* is the resistance peak value after feeding and $R_{bl,i}$ is the baseline resistance, as detected during the zero-air drawn-in cycle, for the sensor *i*. At present, we do not have sufficient information to confirm whether the response of the sensors that gave a positive response (nanocomposite-based sensors) varies with the variation in chemical species and, therefore, whether this information can be used in ML algorithms. Consequently, we used the variation in the absolute value of the sensor values from the baseline. The duration of the peak phase varies as a function of sample concentrations, ranging from 1 min to 80 s, so that 6–8 data points were considered for each measurement cycle.

In Table 1, the characteristics of the resulting dataset are shown.

The averages and standard deviations for each class are 44 ouE/m^3^ and 36 ouE/m^3^, for Class 0; 518 ouE/m^3^ and 517 ouE/m^3^, for Class 1; 184 ouE/m^3^ and 169 ouE/m^3^, for Class 2; and 416 ouE/m^3^ and 331 ouE/m^3^, for Class 3.

### 2.4. Algorithms for Odor Classification and Regression

MATLAB R2017b (MathWorks, Natick, MA, USA) and Python 3.7 with the packages scikit-learn 0.23 (https://scikit-learn.org/stable/ (accessed on 24 March 2021)) and Keras 2.4 (https://keras.io (accessed on 14 April 2021)) were used for data pretreatment, feature extraction, and model selection. In particular, Keras 2.4 was employed for the MLP model, whereas scikit-learn was used for Random Forest.

#### 2.4.1. Feature Extraction and General Workflow

**Figure 3:**
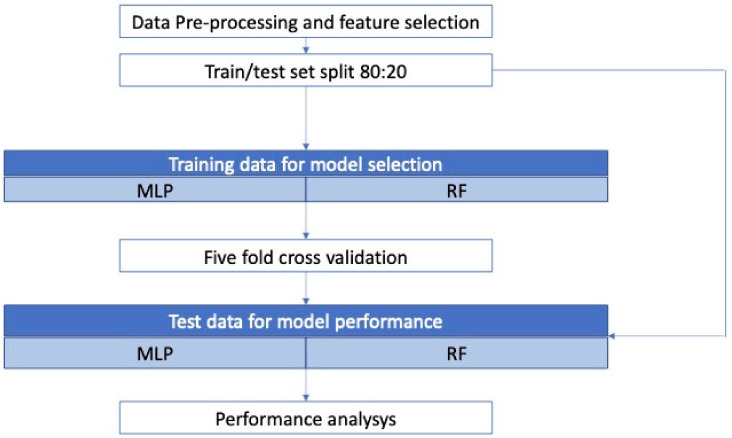
Workflow of model development for the classification and regression tasks.

In Figure 3, the overall workflow for model development is given. After data pretreatment, several tests were carried out to choose an appropriate subset of the input variables—the 32 values of fractional difference *x_i_*—using the Recursive Feature Elimination algorithm with cross-validation (RFECV) [40], such that only the most significant sensors were employed for the specific case. RFECV was employed for both the classification and the regression task.

The overall dataset with the selected features was split into a training and test set according to the ratio 80:20, so that 600 data points were used for training and 150 were used for testing.

Cross-validation allows one to check for overfitting and is particularly useful when dealing with MLP. A fivefold cross-validation approach was employed for the selected model: at every iteration, the training set (600 data points) was split into five, with one portion being kept as the validation set to test the performance with the trained parameters.

After the cross-validation and selection of the model, the last step was to use the test set (20% of the dataset) to analyze the performances of the selected models for classification and regression problems.

For the classification task, a confusion matrix *CM* was calculated for MLP and RF, and the related scoring parameters were calculated, such as the classification accuracy rate for each class (*ac_i_*) and the overall accuracy rate (*ac_o_*):

(2) $$ac_{i}=\frac{cm_{ii}}{cm_{ii}+\sum_{k\neq i}cm_{ik}+\sum_{j\neq i}cm_{ji}}$$

(3) $$ac_{o}=\frac{\sum_{i}cm_{ii}}{\sum_{i,j}cm_{ij}}$$

where *cm_ij_* are the components of the confusion matrix *CM*.

Moreover, as we were dealing with a multiclassification problem with potentially unbalanced classes—in which Class 0 would be more frequent during real-time acquisition—Cohen’s kappa was used as a score parameter to compare the classification accuracy of the models [41]:

(4) $$k=\frac{Pr\left(a\right)-Pr\left(e\right)}{1-Pr\left(e\right)}$$

where $Pr\left(a\right)$ is the probability of agreement, while $Pr\left(e\right)$ is the measure of the agreement between the model predictions and the actual class values as if happening by chance. The introduction of this parameter is related to the predicted field applications of the IOMS. If, for example, after a monitoring campaign lasting a time *T*, it turns out that the classification gives 90% of *T* for Class 0 and 10% of *T* of Class 1, and we are interested in comparing the predicted classes with the actual ones, using the overall accuracy rate *ac_o_* would distort the result, as it does not take into account that the classes are unbalanced.

Therefore, Cohen’s kappa is used to deal with such situations, which will most likely occur during field monitoring. Furthermore, as Cohen’s kappa exploits the Expected Accuracy, namely, a measure representing the dependence obtained by chance between the predicted and the true classification measure [42], this allows for the comparison of different models, which was one of the goals of the paper.

For regression task, the coefficient of determination (R^2^), and the root mean square error (RMSE) were calculated, which is discussed in Section 3.3.

#### 2.4.2. Artificial Neural Network

In the present work, a feed forward neural network or Multilayer Perceptron (MLP) was employed [43]. ANN models have two contour layers: a first layer or input layer in which the features selected by the RFECV are introduced, and an output layer in which the predicted values are generated: target outputs were the four odor classes for the classification problem and the odor concentrations for the regression. Between these two layers exist one or more layers called hidden layer or layers: when more than one hidden layer is employed, the learning process of neural networks is defined as deep learning. During the neural training phase, different parameters minimize the errors between the input and the predicted variable. To find the best model, it is necessary to use the trial-and-error approach through which different topologies are analyzed. The number of hidden layers and the number of neurons for each layer were selected using the following procedure:

- First, a single hidden layer was employed, the accuracy was calculated, and then other layers were added until the appropriate accuracy was obtained;
- In order to further increase the accuracy, an increasing number of neurons per layer was tested, starting from 10.

The hidden layer activation function selected was *ReLU* as a result of its speed in training. The *sotftmax* output layer activation function was chosen for classification, restricting the output of the model to the range (0,1), while the linear function was chosen for regression.

Two kinds of loss functions for classification and regression were tested, namely, categorical cross entropy and mean squared error. Categorical cross-entropy loss [44] measures the performance of a classification model whose output is a probability value between 0 and 1, as given by the *softmax* function of the output layer. Cross-entropy loss increases as the predicted probability diverges from the actual label, while, on the other hand, a perfect model would have a log cross-entropy loss of 0. Categorical cross-entropy can be used for the classification task but not for regression. On the other hand, mean squared error (MSE) as the loss function can used for both classification and regression; thus, it was chosen after some preliminary tests.

#### 2.4.3. Random Forest

RF is a supervised learning model, i.e., it is an evolution of decision tree learning in which each node focuses on a certain attribute of the data and determines what class (classification) or value (regression) the data are most likely to have [45]. RF was recently employed for odor classification to evaluate product quality [20,46], while no applications for environmental monitoring of odor emissions are known to date; hence, a detailed discussion on the algorithm and its genesis is provided below.

A decision tree consists of a collection of nodes, divided into root nodes, decision nodes, and leaf nodes. The training dataset is passed from the root to leaves, and for each node, there are splitting rules for one specific feature. The goal of each split in a decision tree is to move from a confused dataset to two (or more) purer subsets.

Entropy can be used to measure information disorder. In a classification problem, if we have a dataset with *S* elements divided in *k* classes, the frequency *p_i_* of a class *i* in the dataset is given by *S_i_/S*, where *S_i_* is the number of elements belonging to the class *i*. Then, the information entropy *H_s_* is defined as follows:

(5) $$H_{s}:=-\overset{k}{\sum_{i=1}}p_{i}log_{2}p_{i}.$$

It can be observed that entropy is minimum (=0) if all the elements of *S* belong to the same class *i*, and is maximum ($=log_{2}k$) if all the *k* classes are represented in *S* by the same number of elements. In order to measure how good a split in a decision tree is, one parameter that can be adopted is the Information Gain (*IG*), which represents the difference in entropy before and after the split and is defined as

(6) $$IG:=H_{s}-\overset{r}{\sum_{j=1}}p_{j}H_{s_{j}}$$

where the first term is the entropy before the split, the second term is the entropy after the split, calculated as the sum of the entropy of each *j* class of the *r* subclasses generated by the split, weighted on the frequency *p_j_* of a class *j*. Consequently, at each step, the data are split in a way to obtain the highest value in *IG*, as this leads to the purest subsets.

The aforementioned definitions were clearly developed for the classification tasks, where entropy measures the quality of the correct attribution of an element in the appropriate category or class. For regression, the goal of the RF (as in all the ML methods) is to optimize the prediction error, in the form of Mean Squared Error. Such concepts, initially developed for decision tree learning, are the same in RF, which is a model that takes into account multiple decision tree models developed from random subsets of the training data. Bootstrap is the technique that involves random sampling of small subset of data from the training dataset. In comparison with a single decision tree, an RF model achieves better precision values. The way in which the multiple randomly selected decision trees cooperate to give the predictions is called bootstrap aggregation or bagging. Bagging is an ensemble technique that is used to reduce the variance of the prediction model by combining the results of multiple decision trees modeled on different subsets of instances of the dataset. During classification or regression on new data, each tree “votes” by contributing its own prediction to the ensemble’s final prediction; in classification, the class with the most votes is the final prediction, whereas the ensemble average of the trees is the final prediction for regression. The number of trees employed in RF is usually called the number of estimators.

To find the best RF model, the following procedure was adopted: the accuracies for different random forests were calculated with a number of trees (estimators) between 1 and 100, and the RF structure that obtained the best accuracy was chosen.

## 3. Results and Discussion

### 3.1. Feature Selection

In order to select the independent variables that best reflect the input and output relationship and remove the redundant independent variables, the Recursive Feature Elimination algorithm with cross-validation (RFECV) was adopted for selecting a subset of the 32 values of fractional difference *x_i_*, which was used as input to the models. It was decided to use features that were between rank 1 and rank 8 of the RFECV algorithm to have a balance between the number of selected features, which we set to 12. Features selected for classification based off the RFECV ranking were the following sensors: S13 (MOS), S21 (NCA), S24 (NCA), S44 (MOS), S46 (MOS), S7 (EC), S57 (MOS), S54 (NCA), S11 (MOS), S42 (MOS), S23 (NCA), and S9 (EC). Features selected for regression based off the RFECV ranking were the following sensors: S12 (MOS), S5 (AS), S55 (NCA), S11 (MOS), S49 (MOS), S47 (MOS), S7 (EC), S10 (EC), S4 (AS), S51 (NCA), S13 (MOS), and S23 (NCA). As a result of the nature of the RFECV algorithm, it is not straightforward to explain the differences in the lists between classification and regression. However, it can be pointed out that Environmental sensors S5 and S4 were relevant when the regression task was to be carried out by the IOMS, while it appears that classification was not affected by such conditions. This observation is consistent with the literature [13], wherein classification was carried out using 13 MOS chemical sensors (MOS), without taking into account the three environmental sensors; however, more recent findings [47] suggest that the temperature of the gaseous flux in the measurement chamber can be considered as a relevant feature in order to increase the overall classification accuracy (from 96% to 98%).

### 3.2. Classification

**Figure 4:**
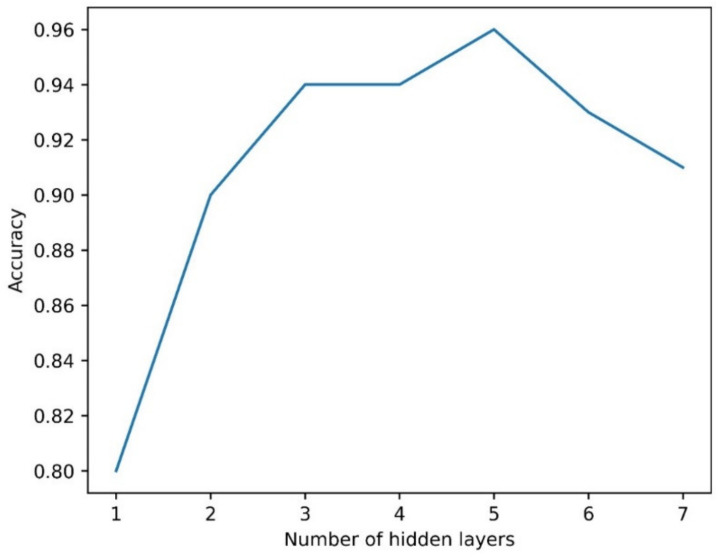
MLP: accuracy in relation to the number of hidden layers.

The hyperparameters of the ML models were selected for the classification task and are represented by the number of hidden layers and the node for MLP and the number of decision trees or estimators for Random Forest. It was decided to increase the number of hidden layers, varying them between 1 and 7, with an initial number of neurons equal to 10. As highlighted in Figure 4, the maximum accuracy for MLP was achieved with five hidden layers.

After selecting the number of hidden layers, a trial-and-error approach was used to select the number of neurons per layer, starting from 10. The best results in terms of accuracy were obtained with 100 neurons.

**Figure 5:**
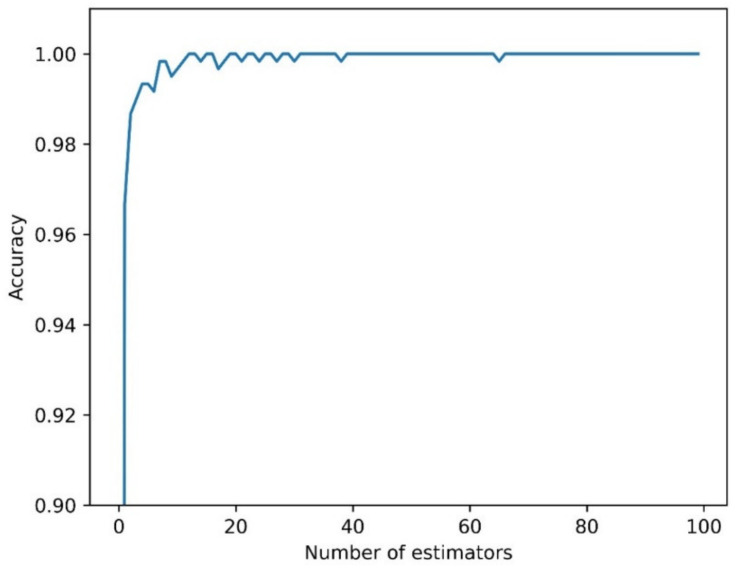
Accuracy in relation to the number of estimators (trees) for Random Forest.

The classification task with RF was first carried out by selecting the hyperparameter of the model, namely, the number of estimators. As can be seen from Figure 5, the accuracy value for RF rapidly increased and stabilized when more than five estimators were used; thus, 20 estimators were chosen.

Once the models (MLP and RF) were selected, the classification accuracy rates for each class and the overall accuracy rate were calculated for the best models. The results for the training set are shown in Table 2.

It is not unusual for such high values to occur when testing a model on the training set. To find out if there was an overfitting problem, and consequent poor predictive performances on new data, 5-fold cross-validation (CV) was adopted, which has not been applied thus far in papers dealing with IOMS classification or regression applications in complex field situations. In detail, the overall accuracy rate of MLP and RF was calculated five consecutive times by splitting the training dataset (600 data) into *internal* training data (480 data points) and validation data (120 data points) with different splits each time. The models were fitted on *internal* training data, and the scores were computed with reference to validation data.

Table 3 presents the overall classification accuracies as the scores of the models calculated for each split and the mean cross-validation (CV) score and the associated standard deviation. Low standard deviations indicate that the choice of training data did not affect the overall classification accuracy, thus indicating that overfitting was avoided.

Then, the prediction capability of the selected models was evaluated with the test dataset. The goodness of the classification can be examined through the construction of a confusion matrix through which we observe which odor classes have been correctly classified and to what quantity. Each row of the matrix represents the instances in an actual class, while each column represents the instances that the classifier assigns.

**Figure 6:**
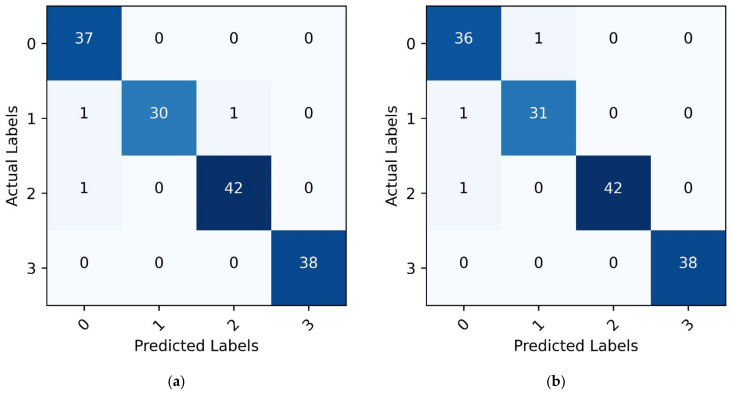
Confusion matrix for the test dataset with two classification algorithms: (**a**) MLP; (**b**) Random Forest.

Figure 6 shows the confusion matrix obtained by MLP and Random Forest for the test dataset.

As regards the overall result, it can be seen that only three elements out of 150 were mismatched for both MLP and RF. By constructing the confusion matrix, the classification accuracy rates, the overall accuracy rate, and Cohen’s kappa can be easily calculated, as $Pr\left(a\right)$ is given by the sum of the diagonal elements in the confusion matrix divided by the total, whereas the $Pr\left(e\right)$ definition is more complex, but it can be recovered from the components of the confusion matrix.

In Table 4, the aforementioned classification metrics are shown for the test dataset. The Cohen’s kappa coefficient, which is used in the present paper as a suitable score parameter for multiclass classification, is 97% for RF and MLP.

Apart from the overall accuracy rates, it is interesting to highlight the classification rate for Class 0, which represents ambient air without the influence of odor emissions from the three sources considered in the study, i.e., pretreatments (Class 1), sludge conditioning (Class 2), and biogas from anaerobic digestion (Class 3). No difference was observed in the classification accuracy rate between Class 0 and the other classes.

In a recent IOMS field application, misclassification of ambient air was found for both the algorithms employed (Linear Discriminant Analysis and ANN), which were not able to identify this class in any case. It was suggested that this was because the sensors were more sensitive to odorous classes [13]. In the present case, ANN performed reasonably well for Class 0, giving a specific classification accuracy of 98% for this class, as compared to poor values obtained in [13], in which one hidden layer was chosen, as opposed to five in the present work. The divergence can be partially attributed to the different models, as in the last few years, it was recognized that a deeper model provides a hierarchy of layers that builds up increasing levels of abstraction from the space of the input variables to the output variables, thus suggesting that using deep architectures expresses a useful prior over the space functions the model learns [48]. Furthermore, RF also performed well for Class 0, so that the significant difference in the classification accuracy rate might be related to the different characteristics of the ambient air, which, in our case, was not totally odorless as a result of the presence of another wastewater treatment plant close by, emitting odorous compounds very different from the investigated classes. It is probable that the presence of other external sources in the ambient air was helpful for better discriminating the WWTP sources. This hypothesis could have been tested by sampling the odor emissions of the outer sources, but unfortunately, this was not possible. Access to each emission source, in fact, is essential to ascertain whether the Class 0 detections (not recognized) refer to emissions from another source or to ambient odorless air. This must be taken into account when IOMS are to be installed outside the fenceline of industrial emission sources, when they are not available for sampling. In other words, the current state of knowledge suggests that is not meaningful to use an IOMS for classification when all the relevant odor emission sources are not sampled and used for training.

### 3.3. Regression

First, the results obtained on the training set are discussed; then, the results from the 5-fold cross-validation and the performance of the models measured on the test set are shown. For odor concentration regression, we chose the same MLP structure as the classification, and the associated results turned out to be satisfying. Table 5 shows the coefficient of determination (R^2^) and root mean squared error (RMSE) for both models based on the training dataset. We also attempted to reduce the number of hidden layers and neurons to obtain a simpler network, but this only led to a loss of performance similar to that seen in classification (Figure 4), with no significant decrease in terms of computational time; thus, there was no advantage in changing network hyperparameters.

Both algorithms were found to be very precise on the training set with equal R^2^. In a similar case concerning odor emissions from WWTP [30], a similar value of R^2^ (0.996) was found for the training set, with an ANN model (13 nodes for input layer, one hidden layer, with eight neurons) developed on data provided by seedOA IOMS [13]. RMSE was equal to 36.9 ouE/m^3^ for MLP and 6.8 ouE/m^3^ for RF, so that RF may be considered more accurate than MLP, based on the training dataset.

Keeping in mind the results of [30], a straightforward comparison of the RMSE is not possible, as the dependent variable (odor concentration) ranged from 20 to 2435 ouE/m^3^ in our case, while it varied from 20 to 50,000 ouE/m^3^ in [30]. By normalizing the RMSE using the difference between the maximum (y_max_) and minimum (y_min_) of the training dataset (NRMSE = RMSE/(y_max_ − y_min_)), we obtained similar results for MLP and ANN in [30], with an NRMSE between 1.05% and 1.52%, while RF demonstrated an NRMSE of 0.28%.

**Figure 7:**
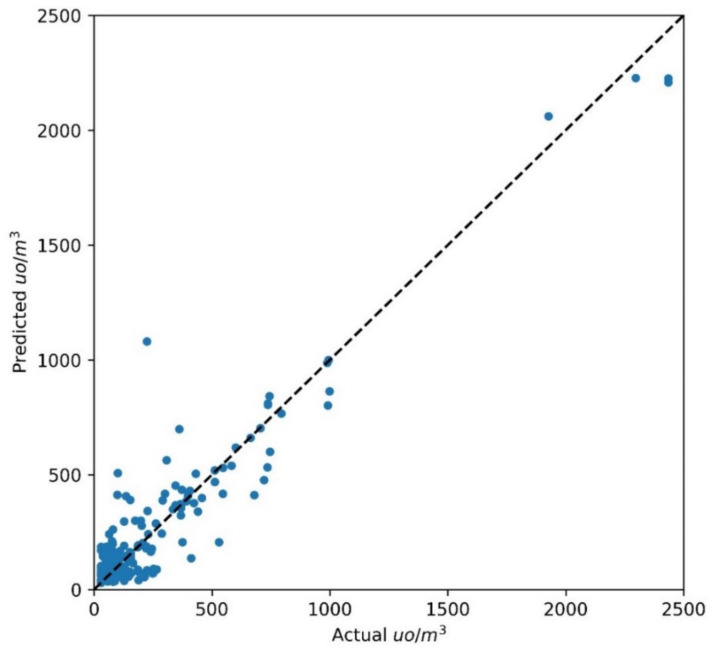
Actual and predicted values of odor concentration (ou/m^3^) obtained with MLP on the test set.

For the regression task, 5-fold cross-validation was also adopted to control whether the proposed models were overfitted and then could have poor predictive performance on new data. The mean CV score (R^2^) and standard deviation CV score for the models were 0.9 and 0.008 for MLP and 0.95 and 0.029 for RF, respectively. Low standard deviations for the cross-validation scores for both models suggest that overfitting was avoided and the models could be used for prediction on the test set. In Figure 7, the correlation between the odor concentrations measured using dynamic olfactometry for the test set (150 data points) and the values predicted by MLP is shown.

**Figure 8:**
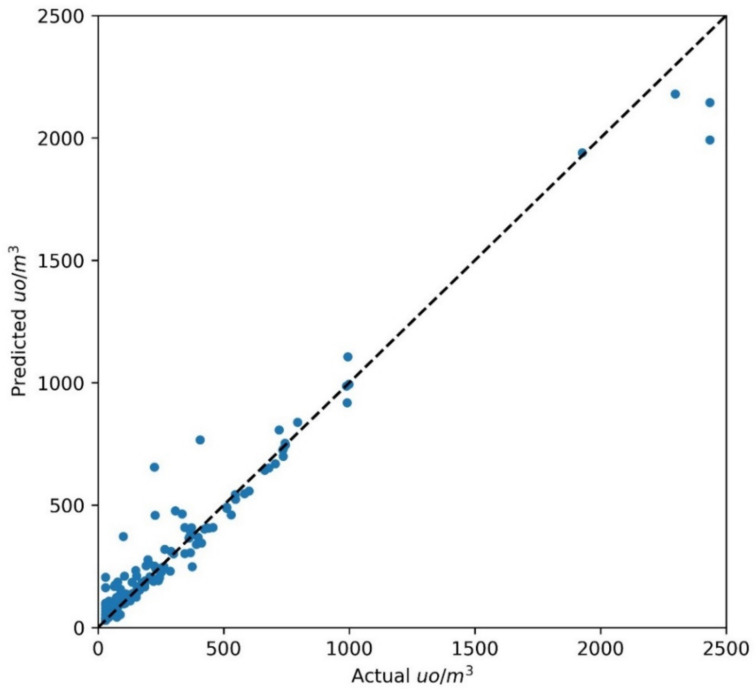
Actual and predicted values of odor concentration (ou/m^3^) obtained with RF on the test set.

The coefficient of determination on the test set was 0.9, while the RMSE was 130 ouE/m^3^, which is greater than the RMSE calculated on the training set (36.9 ouE/m^3^). The NRMSE for MLP on the test data was 5.37%, which can be considered satisfying if we consider the uncertainty associated with measures with dynamic olfactometry [49]. The Random Forest model demonstrated comparable or slightly better results than MLP on the training set, and this was confirmed for the test set, as indicated in Figure 8.

The coefficient of determination on the test set was 0.92, while the RMSE was 97 ouE/m^3^: ten times greater than the RMSE calculated on the training set. Unfortunately, no performance indicators on the test set are available from [30], so that in Table 6, a straightforward comparison between the models is only possible for those proposed in the present work.

While the two different algorithms, i.e., MLP and RF, exhibited almost the same performance for the classification, for the regression, we obtained slightly different values, which may be due to several factors. These include the difference between the RF classifier and RF regressor, the different features selected by the feature selection algorithm for classification and regression, and the different types of targets for regression (continuous variable) and classification (discrete variable). Although the NRMSE for RF on the test data was 4%, indicating a better overall performance than MLP, it must be pointed out that the incremental ratio in the RMSE between the training set and test set was 3.5, whereas a 14-fold increase was observed when comparing the RMSE for the training set (6.8 ouE/m^3^) and the test set (97 ouE/m^3^) for RF. Collecting more samples from different WWTP plants [29] in order to increase the size of the dataset will be a valuable approach to confirm the discrepancy between the RMSE in the training set and test set and whether the NRMSE from the test set is below 10%. These can be considered good results for reliable continuous fenceline monitoring of odor emissions.

The results show that it is possible, even for complex situations, to develop field instrumental odor monitoring applications enhanced by ML algorithms, which are capable of simultaneously performing classification and regression, with interesting practical applications. In fact, it is thought that the dissemination of detailed knowledge concerning algorithms and their performance in the environmental monitoring of odors, with transparent protocols useful for verifying their performance, will make policy makers and environmental protection agencies increasingly inclined to set odor thresholds at plant fencelines [33] and evaluate these values in a monitoring campaign. This has already happened in recent years [21,22,28,29] due to growing pressure from the public and citizens’ complaints. In such a rapidly evolving context, it may be useful to carry out classification and regression simultaneously from both the plant operator’s and the Environmental Protection Agency’s point of view. When fence monitoring with IOMS is mandatory or strongly encouraged, plant operators will be interested in establishing the source of odor emission within a plant that is responsible for the highest concentrations detected at the fenceline, especially when the sources cannot be directly monitored, as in the case of fugitive emissions. If, on the other hand, the Environmental Protection Agency is requested to identify the most critical emission sources from one or more plants located nearby, concentration alone or odor class alone is insufficient to carry out this task; i.e., both odor concentration and class are essential to tackle such complex tasks.

In the present case study, it may be necessary to establish the distribution of odor classes (0, 1, 2, 3) within a specific concentration range. If odor Class 0 (ambient air) is more frequent at low odor concentrations, this odor class is going to be associated with clean background air, while if odor Class 0 is more frequent at high concentrations, another source will be responsible for high odor concentrations. This source may have come be from outside the plant, having not been identified during IOMS training. The monitoring campaign to be carried out in the WWTP of Monopoli will address such issues. Although it is not possible to carry out classification when all odor emission sources are not used for training (Section 3.2), the joint analysis of concentrations and odor classes could provide useful information on complex multisource odor emission problems.

## 4. Conclusions

The paper describes the employment of two machine learning algorithms (Multilayer Perceptron and Random Forest) to elaborate signals acquired from a commercial IOMS used for fenceline odor monitoring at an urban wastewater treatment plant, in order to develop concurrent odor classification and regression models.

As for classification with multiple odor classes, Cohen’s kappa was used as the score parameter to measure the performances of the algorithms on the test set, yielding 97% for both models. For odor concentration regression, NRMSE was used as the performance parameter by normalizing RMSE using the difference between the maximum and minimum of the odor concentrations range. This was done in order to obtain a performance parameter useful for directly comparing the accuracies of IOMSs, even those operating in real cases with different odor emission intensities.

The research aimed to support machine learning applied to environmental odor monitoring with IOMS. In the near future, low-cost sensors using specific performance factors (Cohen’s kappa for classification and NRMSE for regression) will facilitate the use of such systems in the environmental monitoring sector and ensure the possibility of performance comparison between different IOMS.

## Figures and Tables

**Table 1:** Structure of the dataset used, divided by odor classes and indicating the odor concentrations range.

Classes	Number of Samples ^1^	Number of Data	Concentrations Range [ouE/m^3^]
Class 0	28	216	20–200
Class 1	24	160	25–2435
Class 2	28	214	40–510
Class 3	24	160	64–1866

^1^ Including replicates.

**Table 2:** Classification accuracy rates for each class and the overall accuracy rates for MLP and RF with the training dataset.

Classes	MLP	RF
Class 0	0.99	1
Class 1	0.99	1
Class 2	1	1
Class 3	1	1
Overall	0.99	1

**Table 3:** Five-fold cross-validation results.

	Overall Classification Accuracy	
	MLP	RF
First Split	0.95	0.97
Second Split	0.98	0.98
Third Split	0.97	0.97
Fourth Split	0.98	0.99
Fifth Split	0.97	0.97
Mean CV Score	0.97	0.98
Standard Deviation CV Score	0.05	0.03

**Table 4:** Classification accuracy rates for each class, overall accuracy rates, and Cohen’s kappa for MLP and RF with the test dataset.

Classes	MLP	RF
Class 0	0.98	0.97
Class 1	0.98	0.98
Class 2	0.98	0.99
Class 3	1	1
Overall Accuracy Rate	0.98	0.98
Cohen’s Kappa	0.97	0.97

**Table 5:** Regression coefficients and root mean square error for MLP, RF, and ANN developed in [30], based on the training dataset.

Classes	MLP	RF	ANN [30]
R^2^	0.99	0.99	0.9976
RMSE (ouE/m^3^)	36.9	6.8	523,4
NRMSE (%)	1.52	0.28	1.05

**Table 6:** Regression coefficients and root mean square error for MLP and RF based on the test dataset.

Classes	MLP	RF
R^2^	0.9	0.92
RMSE (ouE/m^3^)	130	97
NRMSE (%)	5.37	4.02

## Data Availability

Data available on request due to restrictions.

## References

[1] Hayes J.E., Stevenson R.J., Stuetz R.M. (2014). The impact of malodour on communities: A review of assessment techniques. Sci. Total Environ..

[2] Bokowa A., Diaz C., Koziel J.A., McGinley M., Barclay J., Schauberger G., Guillot J.-M., Sneath R., Capelli L., Zorich V. (2021). Summary and overview of the odour regulations worldwide. Atmosphere.

[3] General Determinations Regarding the Characterization of Atmospheric Emissions from Activities with a High Odour Impact, D.g.r. 15 February 2012–n. IX/3018. http://www.olfattometria.com/download/dgr-lomb.pdf.

[4] Law on Odour Emissions. L.R. 16 July 2018–n. 32. http://www.ager.puglia.it/documents/10192/29519220/LR_32_2018.pdf.

[5] Brattoli M., Mazzone A., Giua R., Assennato G., de Gennaro G. (2016). Automated Collection of Real-Time Alerts of Citizens as a Useful Tool to Continuously Monitor Malodorous Emissions. Int. J. Environ. Res. Pub. Health.

[6] Real Time, Automatic and Remote-Activated Sampling System for Industrial Odour Emissions Compliant with the European Standard EN 13725, CORDIS EU Research Results. cordis.europa.eu/project/id/756865.

[7] Lotesoriere B., Giacomello A., Bax C., Capelli L. (2021). The Italian Pilot Study of the D-NOSES Project: An Integrated Approach Involving Citizen Science and Olfactometry to Identify Odour Sources in the Area of Castellanza (VA). Chem. Eng. Trans..

[8] Karakaya D., Ulucan O., Turkan M. (2020). Electronic Nose and Its Applications: A Survey. Int. J. Aut. Comp..

[9] Stuetz R.M., Fenner R.A., Engin G. (1999). Assessment of odours from sewage treatment works by an electronic nose, H_2_S analyzer and olfactometry. Water Res..

[10] Qu G., Omotoso M.M., el-Din M.G., Feddes J.J.R. (2008). Development of an integrated sensor to measure odors. Environ. Monit. Assess..

[11] Bax C., Sironi S., Capelli L. (2020). How Can Odors Be Measured? An Overview of Methods and Their Applications. Atmosphere.

[12] Yan J., Guo X., Duan S., Jia P., Wang L., Peng C., Zhang S. (2015). Electronic Nose Feature Extraction Methods: A Review. Sensors.

[13] Zarra T., Galang M.G.K., Ballesteros F.C., Belgiorno V., Naddeo V. (2021). Instrumental Odour Monitoring System Classification Performance Optimization by Analysis of Different Pattern-Recognition and Feature Extraction Techniques. Sensors.

[14] Vanarse A., Espinosa-Ramos J.I., Osseiran A., Rassau A., Kasabov N. (2020). Application of a Brain-Inspired Spiking Neural Network Architecture to Odor Data Classification. Sensors.

[15] Wen T., Mo Z., Li J., Liu Q., Wu L., Luo D. (2021). An Odor Labeling Convolutional Encoder–Decoder for Odor Sensing in Machine Olfaction. Sensors.

[16] Yan L., Wu C., Liu J. (2020). Visual Analysis of Odor Interaction Based on Support Vector Regression Method. Sensors.

[17] Misselbrook T.H., Hobbs P.J., Persaud K.C. (1997). Use of an Electronic Nose to Measure Odour Concentration Following Application of Cattle Slurry to Grassland. J. Agric. Eng. Res..

[18] Aguilera T., Lozano J., Paredes J., Álvarez F., Suárez J. (2012). Electronic nose based on independent component analysis combined with partial least squares and artificial neural networks for wine prediction. Sensors.

[19] Zhang L., Tian F., Nie H., Dang L., Li G., Ye Q., Kadri C. (2012). Classification of multiple indoor air contaminants by an electronic nose and a hybrid support vector machine. Sens. Actuators B Chem..

[20] Men H., Fu S., Yang J., Cheng M., Shi Y., Liu J. (2018). Comparison of SVM, RF and ELM on an Electronic Nose for the Intelligent Evaluation of Paraffin Samples. Sensors.

[21] Cangialosi F., Intini G., Colucci D. (2018). On Line Monitoring of Odour Nuisance at a Sanitary Landfill for Non-Hazardous Waste. Chem. Eng. Trans..

[22] Bax C., Lotesoriere B., Capelli L. (2021). Real-time Monitoring of Odour Concentration at a Landfill Fenceline: Performance Verification in the Field. Chem. Eng. Trans..

[23] Cangialosi F. (2021). Advanced Data Mining for Odour Emissions Monitoring: Experimental Peak-to-mean Calculations and Spectral Analysis of Data Derived from Ioms in two waste Treatment Plants. Chem. Eng. Trans..

[24] Majchrzak T., Wojnowski W., Dymerski T., Gębicki J., Namieśnik J. (2018). Electronic noses in classification and quality control of edible oils: A review. Food Chem..

[25] Tan J., Xu J. (2020). Applications of electronic nose (e-nose) and electronic tongue (e-tongue) in food quality-related properties determination: A. review. Artific. Intell. Agric..

[26] Wijaya D.R., Sarno R., Zulaika E. (2021). DWTLSTM for electronic nose signal processing in beef quality monitoring. Sens. Actuators B Chem..

[27] Bax C., Sironi S., Capelli L. (2020). Definition and Application of a Protocol for Electronic Nose Field Performance Testing: Example of Odor Monitoring from a Tire Storage Area. Atmosphere.

[28] Oliva G., Zarra T., Pittoni V., Senatore V., Galang M.G.M., Castellani M., Belgiorno V., Naddeo V. (2021). Next-generation of instrumental odour monitoring system (IOMS) for the gaseous emissions control in complex industrial plants. Chemosphere.

[29] Research Project ASPIDI. https://www.progettoaspidi.com.

[30] Galang M.G., Zarra T., Naddeo V., Belgiorno V., Ballesteros F.C. (2018). Artificial Neural Network in the Measurement of Environmental Odours by E-Nose. Chem. Eng. Trans..

[31] Zarra T., Galang M.G., Ballesteros F., Naddeo V., Belgiorno V. (2019). Environmental odour management by artificial neural network—A review. Environ. Int..

[32] Naddeo V., Zarra T., Oliva G., Kubo A., Ukida N., Higuchi T. (2016). Odour measurement in wastewater treatment plant by a new prototype of e.Nose: Correlation and comparison study with reference to both European and Japanese approaches. Chem. Eng. Trans..

[33] Guidelines for Issuing Technical Opinions Regarding the Emissions into Atmosphere Produced by Wastewater Treatment Plant. ARPA Puglia (rev. 2014). https://old.arpa.puglia.it/c/document_library/get_file?uuid=6e747fc8-859a-4cd6-9302-bb73913f7410&groupId=13879.

[34] UNI 11761:2019; Emissioni e Qualità Dell’aria-Determinazione Degli Odori Tramite IOMS (Instrumental Odour Monitoring Systems). http://store.uni.com/catalogo/uni-11761-2019.

[35] Eusebio L., Capelli L., Sironi S. (2016). Electronic Nose Testing Procedure for the Definition of Minimum Performance Requirements for Environmental Odor Monitoring. Sensors.

[36] Wu Z., Zhang H., Sun W., Lu N., Yan M., Wu Y., Hua Z., Fan S. (2020). Development of a Low-Cost Portable Electronic Nose for Cigarette Brands Identification. Sensors.

[37] Savitzky A., Golay M.J.E. (1965). Smoothing and differentiation of data by simplified least squares procedures. Anal. Chem..

[38] De Oliveira M.A., Araujo N.V.S., Da Silva R.N., Da Silva T.I., Epaarachchi J. (2018). Use of Savitzky–Golay Filter for Performances Improvement of SHM Systems Based on Neural Networks and Distributed PZT Sensors. Sensors.

[39] Stetter J.R., Penrose W.R. (2002). Understanding chemical sensors and chemical sensor arrays (electronic noses): Past, present, and future. Sensors.

[40] Demarchi L., Kania A., Ciężkowski W., Piórkowski H., Oświecimska-Piasko Z., Chormański J. (2020). Recursive Feature Elimination and Random Forest Classification of Natura 2000 Grasslands in Lowland River Valleys of Poland Based on Airborne Hyperspectral and LiDAR Data Fusion. Remote Sens..

[41] Dietterich T.G. (1998). Approximate statistical tests for comparing supervised classification learning algorithms. Neural Comp..

[42] Grandini M., Bagli E., Visani G. Metrics for Multi-Class Classification: An Overview. https://arxiv.org/pdf/2008.05756v1.pdf.

[43] Gardner M.W., Dorling S.R. (1997). Artificial neural networks (the multilayer perception)—A review of applications in the atmospheric sciences. Atmos. Environ..

[44] Zhang Z., Sabuncu M.R. Generalized Cross Entropy Loss for Training Deep Neural Networks with Noisy Labels. Proceedings of the 32nd Conference on Neural Information Processing Systems (NeurIPS 2018).

[45] Breiman L. (2001). Random Forests. Mach. Learn..

[46] Men H., Jiao Y., Shi Y., Gong F., Chen Y., Fang H., Liu J. (2018). Odor Fingerprint Analysis Using Feature Mining Method Based on Olfactory Sensory Evaluation. Sensors.

[47] Oliva G., Zarra T., Massimo R., Senatore V., Buonerba A., Belgiorno V., Naddeo V. (2021). Optimization of Classification Prediction Performances of an Instrumental Odour Monitoring System by Using Temperature Correction Approach. Chemosensors.

[48] Goodfellow I., Bengio Y. (2016). Deep Learning.

[49] Van Harreveld A.P. (2021). Update on the revised EN 13725:2021. Chem. Eng. Trans..

